# Effects of Pharmacological and Agrochemical Endocrine Disruptors on Human Sperm Mitochondrial Respiration: Evidence from Ex Vivo Bioenergetic Profiling

**DOI:** 10.3390/jox16010031

**Published:** 2026-02-09

**Authors:** Graziana Assalve, Paola Lunetti, Vincenzo Zara, Alessandra Ferramosca

**Affiliations:** Department of Experimental Medicine, University of Salento, I-73100 Lecce, Italy; graziana.assalve@unisalento.it (G.A.); paola.lunetti@unisalento.it (P.L.); vincenzo.zara@unisalento.it (V.Z.)

**Keywords:** endocrine-disrupting chemicals, dihydroxyflutamide, bicalutamide, lindane, permethrin, mancozeb, tributyltin oxide, sperm mitochondria

## Abstract

**Background:** Human exposure to endocrine-disrupting chemicals (EDCs) is increasingly linked to male reproductive dysfunction, but underlying mechanisms remain unclear. This study aimed to evaluate how selected pharmacological (dihydroxyflutamide, 2OH-FTA; bicalutamide, BIC) and agrochemical (lindane, βHCH; permethrin, PERM; mancozeb, MNZ; tributyltin oxide, TBTO) EDCs affect mitochondrial function in human spermatozoa with parameters within World Health Organization (WHO) reference ranges. **Methods:** Human sperm cells were exposed ex vivo to 0.1–1000 nM of each compound. Mitochondrial respiration was measured using polarography, assessing oxygen consumption in active (V_3_) and resting (V_4_) states, and the respiratory control ratio (RCR) was calculated as an index of mitochondrial coupling. **Results:** Both 2OH-FTA and BIC reduced RCR in a concentration-dependent manner, mainly due to increases in V_4_, with BIC showing the strongest effect. βHCH produced a similar pattern, elevating V_4_ and decreasing RCR. In contrast, PERM, MNZ, and TBTO caused near-complete collapse of both V_3_ and V_4_ even at sub-nanomolar concentrations, indicating severe, concentration-independent mitochondrial toxicity. **Conclusions:** Sperm mitochondria are highly sensitive to EDCs, and distinct compounds exert different bioenergetic effects. Mitochondrial respiration assays provide a useful tool for ex vivo toxicological screening and risk assessment.

## 1. Introduction

In recent years, growing attention has been directed toward the potential health risks associated with continuous human exposure to endocrine-disrupting chemicals (EDCs), a broad class of compounds primarily derived from anthropogenic activities that are capable of interfering with the physiological regulation of the endocrine system [1].

EDCs comprise a highly heterogeneous group of substances, including insecticides and pesticides (e.g., dichlorodiphenyltrichloroethane, DDT), plasticizers and material additives (e.g., di-2-ethylhexyl phthalate, DEHP), pharmaceuticals used for contraception (e.g., levonorgestrel) or hormone replacement therapy (e.g., synthetic estrogens), as well as naturally occurring phytoestrogens (e.g., genistein), which are predominantly found in legumes and cereals [2,3,4,5].

Human exposure to EDCs occurs through multiple routes, including inhalation of contaminated air and particulate matter, ingestion of polluted food and water, and dermal contact [6]. A major concern related to these compounds is their environmental persistence, which promotes bioaccumulation as a consequence of widespread production, extensive use, and inadequate disposal practices [7,8,9].

Epidemiological and experimental studies have associated EDC exposure with a wide range of adverse health outcomes, such as gynecological disorders, male reproductive dysfunction and increased susceptibility to hormone-dependent cancers, including prostate and breast cancer, as well as cardiovascular, neurological, and metabolic diseases [10,11,12,13,14,15,16,17,18,19,20,21,22,23,24,25].

Particular attention has recently been devoted to the impact of EDCs on male reproductive health, especially in light of the documented global decline in sperm quality, characterized by reduced sperm concentration and decreased circulating testosterone levels [26,27,28]. These trends have been increasingly attributed to chronic exposure to different classes of EDCs [29], among which pharmacological and agrochemical compounds are major contributors [30,31].

EDCs interfere with sex steroid hormone regulation through multiple molecular mechanisms [32], including: (i) mimicking endogenous hormones and activating their receptors (agonistic or mimetic action); (ii) binding to hormone receptors and blocking their physiological activation (antagonistic action); and (iii) altering hormone synthesis, secretion, metabolism, or downstream signaling pathways (triggering action) [33].

Pharmacological EDCs such as dihydroxyflutamide (2OH-FTA) and bicalutamide (BIC) exert antiandrogenic effects by antagonizing androgen receptors, thereby inhibiting androgen-dependent biological processes [34]. 2OH-FTA, an active metabolite of hydroxyflutamide used in prostate cancer therapy, has been shown to impair mitochondrial complexes I, II, and IV, resulting in reduced adenosine triphosphate (ATP) production and decreased sperm motility [35]. Similarly, BIC (also widely employed in prostate cancer treatment) compromises mitochondrial function by downregulating genes encoding respiratory chain complexes, leading to reduced ATP synthesis and increased reactive oxygen species (ROS) production, which ultimately impairs sperm functionality [36].

Agrochemical EDCs, including lindane (βHCH) and mancozeb (MNZ), are likewise known to disrupt male reproductive function and promote infertility by impairing mitochondrial activity and inducing excessive oxidative stress [37,38,39,40,41]. Additional agrochemical EDCs of concern include permethrin (PERM) and tributyltin oxide (TBTO). PERM has been reported to reduce sperm motility in vitro, disrupt mitochondrial membrane integrity, and interfere with testosterone biosynthesis [42,43,44,45]. TBTO, in turn, has been shown to impair spermatogenesis in aquatic organisms, underscoring its potential reproductive toxicity across species [46].

In the present study, we aimed to provide a comprehensive evaluation of the effects of selected pharmacological and agrochemical EDCs on mitochondrial function in human spermatozoa. Using a well-established ex vivo model of sperm mitochondrial respiration [47,48,49], we investigated the impact of 2OH-FTA, BIC, βHCH, PERM, MNZ, and TBTO on sperm mitochondrial bioenergetics in samples meeting the World Health Organization (WHO) reference criteria. Mitochondrial function was assessed through polarographic measurements of oxygen consumption under active (V_3_) and resting (V_4_) respiratory states, as well as by calculating the respiratory control ratio (RCR), a key indicator of the coupling efficiency between electron transport and ATP synthesis. Spermatozoa were exposed to concentrations ranging from 0.1 to 1000 nM, encompassing estimated levels of human dietary and environmental exposure [50,51,52,53,54,55]. Through this functional approach, our study sought to determine whether these EDCs compromise sperm mitochondrial efficiency, thereby providing mechanistic insights into their potential role in male reproductive toxicity and contributing to improved clinical and environmental risk assessment strategies.

## 2. Materials and Methods

### 2.1. Chemicals

All chemicals were purchased from Sigma-Aldrich, St. Louis, MO, USA.

Reduced nicotinamide adenine dinucleotide (NADH, CAS no. 53-84-9) and carbonyl cyanide 4-chlorophenyl hydrazone (CCCP, CAS no. 555-60-2) were used as positive and negative reference controls of the experimental model, respectively, since NADH is a physiological substrate and CCCP is an uncoupler for mitochondrial oxidative phosphorylation (OXPHOS). Dimethyl sulfoxide (DMSO, CAS no. 67-68-5) was used as a blank control for each chemical treatment.

The xenobiotics used in this study (Table 1) included the anticancer drugs 2OH-FTA (CAS no. 52806-53-8) and BIC (CAS no. 90357-06-5), the pesticide βHCH (CAS no. 58-89-9), the insecticide PERM (CAS no. 52645-53-1), the fungicide MNZ (CAS no. 8018-01-7) and the biocide TBTO (CAS no. 56-35-9). Stock solutions of all xenobiotics were prepared in DMSO, in accordance with the manufacturers’ specifications, to ensure complete solubilization.

### 2.2. Male Recruitment and Semen Collection

Semen samples and the corresponding spermiograms used in this study were provided by the biological medical center Tecnomed (Nardò, Lecce, Italy).

The research protocol was reviewed and approved by the Institutional Review Board of the Department of Biological and Environmental Sciences and Technologies, University of Salento (approval date: 27 April 2022), and the study was conducted in full compliance with the principles of the Declaration of Helsinki. All experimental procedures adhered to the applicable guidelines and regulations for research involving human subjects, including Directive 2004/10/EC of the European Parliament and of the Council (11 February 2004) on the application of Good Laboratory Practice principles, as well as the WHO guidelines for semen analysis [56].

Semen samples were obtained from 10 healthy male volunteers with normal body mass index (mean age: 32 years), who provided written informed consent for the use of their semen. Participants reported no medical conditions, lifestyle factors, or pharmacological treatments known to interfere with semen quality. Ejaculates were collected by masturbation after 3–5 days of sexual abstinence and processed within 30 min following complete liquefaction, in accordance with the WHO Laboratory Manual for the Examination and Processing of Human Semen [56]. Sperm motility parameters were evaluated using a computer-assisted sperm analysis system (CASA; SCA^®^ 5.3.0.1, Sperm Class Analyzer, LabIVF Asia Pte Ltd., Singapore).

Semen samples that satisfied the WHO reference criteria for normozoospermia were included in the study. Sperm samples from different donors showing comparable seminal parameters within WHO reference ranges were pooled prior to experimental use. Consequently, all experiments were performed on pooled samples rather than on individual donor samples.

Sperm cells were obtained by centrifugation at 800× *g* for 10 min at room temperature and resuspended in an isotonic salt solution composed of 2 g/L bovine serum albumin (BSA), 113 mM KCl, 12.5 mM KH_2_PO_4_, 2.5 mM K_2_HPO_4_, 3 mM MgCl_2_, 0.4 mM ethylenediaminetetraacetic acid (EDTA), and 20 mM Tris, adjusted to pH 7.4 with HCl, for mitochondrial respiration assays [47].

### 2.3. Human Sperm Exposure to Chemicals

Human spermatozoa were adjusted to a final concentration of 10 × 10^6^ sperm cells/mL and incubated for 1 h at 37 °C with the compounds listed in Table 1 at final concentrations of 0.1, 1, 10, 100, and 1000 nM [48,49].

Control samples (blank, 0 nM) were incubated in medium containing 1% DMSO, which served as the vehicle for all tested chemicals. Each experimental set comprised six parallel conditions (blank and five exposure concentrations) and was independently replicated four times under identical experimental settings.

### 2.4. Mitochondria Respiration Studies

After chemical exposure, spermatozoa were subjected to a hypotonic treatment and subsequently analyzed for mitochondrial respiratory activity by polarographic measurement of oxygen consumption, as previously described [47,48,49].

Oxygen consumption rates were measured using a Clark-type oxygen electrode (Hansatech Oxygraph, King’s Lynn, UK) and expressed as nmol O_2_ × mL^−1^ × min^−1^/(10 × 10^6^ cells).

Measurements were performed in the presence of respiratory substrates (10 mM pyruvate and 10 mM malate) and 0.76 μM adenosine diphosphate (ADP). The rate of oxygen consumption under phosphorylating conditions (V_3_) was determined in the presence of pyruvate, malate, and ADP, whereas the resting respiration rate (V_4_) was measured in the presence of pyruvate and malate alone. Mitochondrial coupling efficiency was evaluated by calculating the respiratory control ratio (RCR), defined as the ratio between V_3_ and V_4_.

### 2.5. Statistics

Statistical analyses were performed using GraphPad Prism version 8.0.2 (GraphPad Software, Inc., La Jolla, CA, USA). All experiments were independently replicated four times under identical experimental conditions, with each replicate corresponding to an independent experimental run performed on pooled sperm samples with comparable seminal characteristics. Data are presented as mean ± standard deviation (SD). Differences among multiple experimental groups were analyzed using two-way analysis of variance (ANOVA), followed by Dunnett’s post hoc test for comparisons between treated samples and the control group. Differences were considered statistically significant at *p* < 0.05.

## 3. Results

### 3.1. Establishment and Validation of the Ex Vivo Assay

To establish and validate the ex vivo experimental system, human spermatozoa were exposed to two reference compounds commonly used to modulate mitochondrial OXPHOS: NADH, employed as a positive control acting as a respiratory substrate, and CCCP, used as a negative control due to its uncoupling activity [48,57].

Their effects were compared with those observed in control samples treated with 1% DMSO, which did not differ significantly from water-treated controls [48].

**Figure 1:**
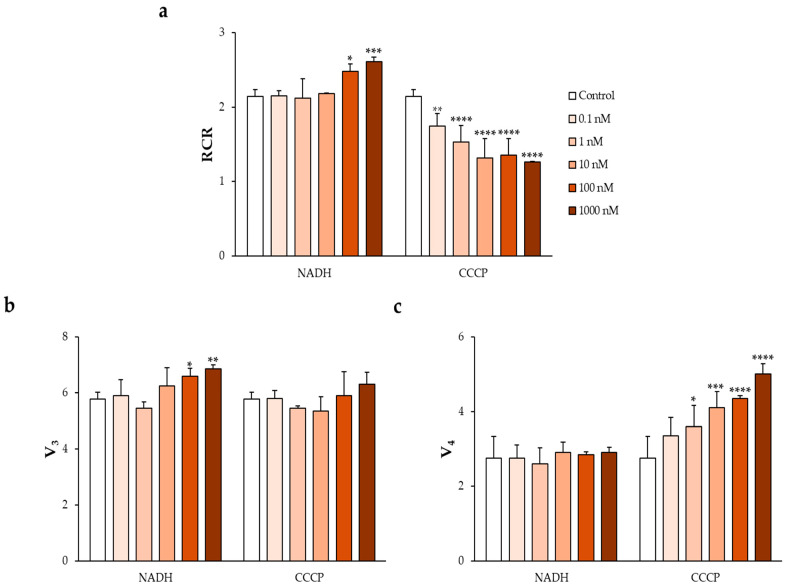
Establishment of the ex vivo model by exposing human sperm samples to increasing concentrations of the reference compounds nicotinamide adenine dinucleotide reduced (NADH) and carbonyl cyanide 4-chlorophenyl hydrazone (CCCP). (**a**) Respiratory control ratio (RCR), together with changes in (**b**) oxygen consumption during the active respiratory state (V_3_) and (**c**) oxygen consumption in the resting state (V_4_), were measured. Data are expressed as mean ± standard deviation (SD). Statistical analysis was performed using two-way analysis of variance (ANOVA) followed by Dunnett’s post hoc test. * *p* < 0.05, ** *p* < 0.005, *** *p* < 0.001 and **** *p* < 0.0001 versus control.

As shown in Figure 1a–c, incubation with increasing concentrations of NADH resulted in a gradual increase in V_3_ in normozoospermic samples, which was reflected in a concentration-dependent increase in the V_3_/V_4_ ratio, corresponding to the RCR.

Conversely, exposure to CCCP produced a dose-dependent increase in V_4_, accompanied by a progressive decrease in the RCR.

### 3.2. Impact of Pharmacological EDCs on Mitochondrial Respiration in Human Spermatozoa

Following validation of the ex vivo experimental model, human spermatozoa were exposed to the pharmacological EDCs investigated in this study, namely 2OH-FTA and BIC.

**Figure 2:**
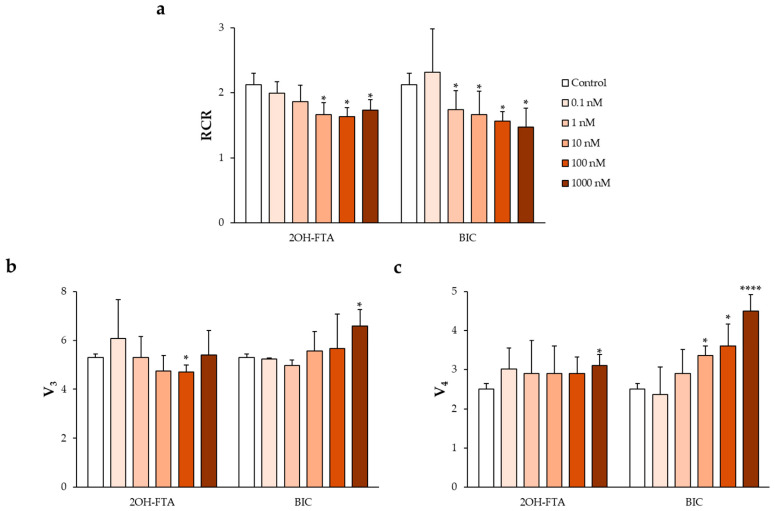
Effects of pharmacological EDCs on mitochondrial respiratory parameters in human spermatozoa. Changes in (**a**) RCR, (**b**) V_3_, and (**c**) V_4_ were evaluated in sperm samples exposed to increasing concentrations of 2OH-FTA and BIC to assess alterations in mitochondrial oxygen consumption and respiratory efficiency. Data are expressed as mean ± SD. Statistical analysis was performed using two-way ANOVA followed by Dunnett’s post hoc test. * *p* < 0.05 and **** *p* < 0.0001 versus control.

Exposure to 2OH-FTA was associated with significant alterations in mitochondrial respiratory parameters. A statistically significant decrease in V_3_ of approximately 11% was observed at 100 nM, while a significant increase in V_4_ of about 24% was detected exclusively at the highest tested concentration (1000 nM) (Figure 2b,c). These changes were accompanied by a significant reduction in the RCR, which reached approximately 20% compared with control values and was observed across a broad concentration range, from 10 to 1000 nM (Figure 2a).

Treatment with BIC also resulted in a significant, concentration-dependent reduction in RCR over the range of 1–1000 nM (Figure 2a). In contrast to 2OH-FTA, BIC exposure led to a progressive increase in V_4_, ranging from approximately 35% at 10 nM to nearly 80% at 1000 nM, while its effects on V_3_ were concentration-dependent, with a decrease of about 6% at 1 nM and a significant increase of approximately 24% at 1000 nM (Figure 2b,c). Overall, the magnitude of the increase in V_4_ exceeded that observed for V_3_, resulting in a net reduction in RCR relative to control samples.

### 3.3. Impact of the Agrochemical EDCs on Mitochondrial Respiration in Human Spermatozoa

Agrochemical EDCs were analyzed individually to assess their effects on mitochondrial respiratory parameters. For comparative purposes, their responses were evaluated alongside those previously reported for the herbicides glyphosate (GLY) and glufosinate ammonium (GA), which have been shown to affect the RCR [48].

**Figure 3:**
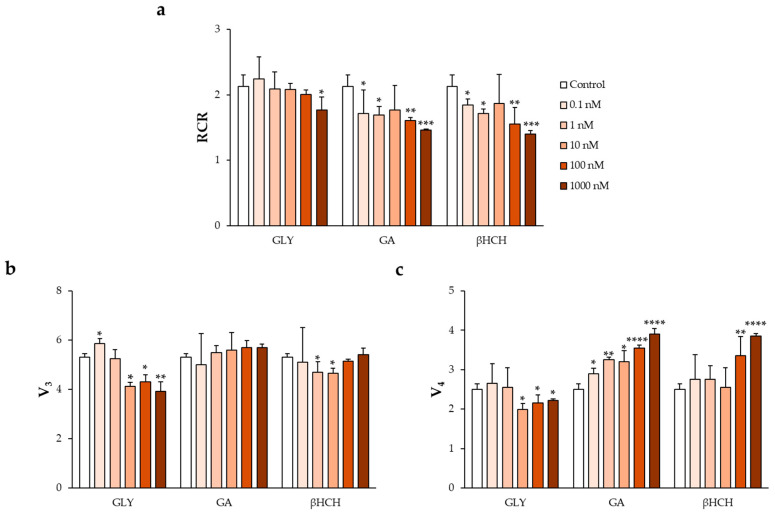
Effects of the agrochemical EDC βHCH on mitochondrial respiratory function in human spermatozoa, compared with the previously characterized herbicides glyphosate (GLY) and glufosinate ammonium (GA). Changes in (**a**) RCR, (**b**) V_3_, and (**c**) V_4_ were evaluated in sperm samples exposed to increasing concentrations of the compounds to assess alterations in mitochondrial oxygen consumption and respiratory efficiency. Data for GLY and GA are included as reference for comparison, based on previously published findings [48]. All values are expressed as mean ± SD. Statistical analysis was performed using two-way ANOVA followed by Dunnett’s post hoc test. * *p* < 0.05, ** *p* < 0.005, *** *p* < 0.001 and **** *p* < 0.0001 versus control.

Among the tested compounds, βHCH induced concentration-dependent alterations in mitochondrial respiration. A reduction in V_3_ of approximately 12% was observed at low concentrations (1 and 10 nM), whereas V_3_ values were comparable to control levels at the highest tested concentration (Figure 3b). In contrast, V_4_ increased by approximately 34% at 100 nM and 54% at 1000 nM (Figure 3c).

These changes were associated with a progressive reduction in RCR, which decreased by approximately 15%, 20%, and 30% across the tested concentration range (Figure 3a).

With respect to the remaining agrochemical EDCs examined, PERM, MNZ, and TBTO, a pronounced reduction in mitochondrial respiratory parameters was detected under the experimental conditions applied. At the lowest concentration tested (0.1 nM), both V_3_ and V_4_ decreased to the minimal detectable value (0.01), and this pattern persisted across all tested concentrations, resulting in an RCR of 1 in each condition (Table 2).

These findings indicate a loss of respiratory coupling and a concentration-independent impairment of mitochondrial function.

## 4. Discussion

Growing evidence identifies EDCs as relevant contributors to male reproductive dysfunction, with sperm quality representing one of the most sensitive biological endpoints affected by environmental and pharmacological exposures. Both antiandrogenic drugs and agrochemical compounds have been associated with impaired spermatogenesis and reduced semen quality, including alterations in sperm concentration, motility, and morphology [30,31,37,38,39,40,44,45]. However, the cellular mechanisms underlying these effects remain incompletely characterized, particularly with respect to mitochondrial bioenergetics in human spermatozoa.

In this study, we investigated the effects of selected pharmacological and agrochemical EDCs on mitochondrial respiration in human sperm samples within the WHO reference ranges. Given the central role of mitochondrial OXPHOS in sustaining sperm motility and fertilizing capacity, mitochondrial respiration represents a highly sensitive and functionally relevant indicator of sperm quality. In this context, it should be noted that the experimental approach adopted in this study is specifically designed to assess mitochondrial bioenergetics independently of plasma membrane-dependent processes. The hypotonic demembranation step required for respiration measurements precludes the reliable evaluation of motility, capacitation status, acrosomal integrity, intracellular pH, calcium signaling, and other membrane-associated functional endpoints, which have been extensively investigated in intact spermatozoa exposed to EDCs [14,37,42,58,59]. By isolating mitochondrial function from membrane-related events, this approach allows a focused mechanistic assessment of mitochondrial vulnerability to EDCs in human spermatozoa.

Consistently, previous studies have demonstrated strong associations between mitochondrial activity, membrane potential, ROS production, and key sperm parameters [60,61,62,63,64], supporting the concept that mitochondria constitute a primary intracellular target of EDC-induced reproductive toxicity [58,59,65].

By focusing on RCR as an index of mitochondrial coupling efficiency, together with the analysis of V_3_ and V_4_, the experimental approach adopted in this study enabled the identification of distinct patterns of mitochondrial dysfunction induced by different classes of EDCs. This analysis provided insight into whether the observed effects were primarily driven by impaired phosphorylating respiration, increased basal oxygen consumption, or a combination of both mechanisms.

Exposure to the pharmacological EDCs 2OH-FTA and BIC resulted in a significant and concentration-dependent reduction in RCR, indicating impaired mitochondrial coupling. Although both compounds ultimately reduced RCR, their respiratory profiles differed. In the case of 2OH-FTA, RCR reduction was associated with a decrease in V_3_ at intermediate concentrations and an increase in V_4_ at higher concentrations, suggesting combined effects on electron transport efficiency and basal respiration. In contrast, BIC exposure was characterized by a marked and progressive increase in V_4_, which exceeded changes in V_3_ and largely accounted for the observed decline in RCR. These findings are consistent with previous reports describing the interference of 2OH-FTA with multiple respiratory chain complexes and the ability of BIC to alter the expression of genes encoding mitochondrial respiratory proteins [35,36]. Such mechanisms provide a plausible link between androgen receptor antagonism and mitochondrial dysfunction in sperm cells.

Among the agrochemical EDCs, βHCH induced a clear dose-dependent reduction in RCR, mainly driven by increased V_4_ values, a profile comparable to that previously reported for GA. This pattern reflects reduced mitochondrial coupling efficiency and is consistent with existing evidence linking pesticide exposure to impaired sperm quality and mitochondrial alterations [37,39,40].

Notably, these effects were observed at nanomolar concentrations, highlighting the high sensitivity of sperm mitochondrial bioenergetics to low-level agrochemical exposure.

In contrast, PERM, MNZ, and TBTO produced a markedly more severe response. For these compounds, both V_3_ and V_4_ collapsed to minimal detectable levels at sub-nanomolar concentrations, resulting in an RCR of 1 across all tested conditions. This uniform response indicates a profound loss of mitochondrial respiratory control and suggests a severe impairment of mitochondrial function that is largely independent of concentration within the tested range. Such a profile is indicative of extensive disruption of mitochondrial bioenergetics and underscores the particularly high mitochondrial toxicity of these agrochemicals in human spermatozoa. Previous studies have associated exposure to these compounds with alterations in sperm morphology, motility, and epigenetic regulation [38,44,45], suggesting that mitochondrial dysfunction may represent a central event underlying their broader reproductive toxicity.

Overall, the results of this study demonstrate that both pharmacological and agrochemical EDCs directly impair mitochondrial bioenergetics in human spermatozoa, albeit through compound-specific patterns and degrees of severity. Given the pivotal role of mitochondria in supporting sperm motility and fertilization competence, mitochondrial dysfunction emerges as a plausible mechanistic link between EDC exposure and reduced male fertility. These findings further support the use of sperm mitochondrial respiration as a sensitive functional endpoint for reproductive toxicity assessment and highlight the importance of incorporating mitochondrial targets into environmental and clinical risk evaluation strategies.

## 5. Conclusions

This study demonstrates that both pharmacological and agrochemical EDCs impair mitochondrial bioenergetics in human spermatozoa at nanomolar concentrations, with compound-specific patterns of toxicity. The anti-androgenic compounds 2OH-FTA and BIC primarily reduced mitochondrial coupling efficiency through increased V_4_, whereas the agrochemical βHCH induced a GA-like respiratory profile characterized by a dose-dependent reduction in RCR.

In contrast, PERM, MNZ, and TBTO caused a profound and concentration-independent suppression of mitochondrial respiration, with both V_3_ and V_4_ reduced to minimal detectable levels even at sub-nanomolar concentrations.

Overall, these findings identify sperm mitochondria as a highly sensitive target of EDC exposure and support the use of mitochondrial respiration assays as a robust ex vivo tool for mechanistic studies and reproductive toxicity risk assessment.

## Figures and Tables

**Table 1:** Substances tested and their classification as endocrine-disrupting chemicals (EDCs).

EDC Class	Chemical	Type of Substance	References
Pharmacological	Dihydroxyflutamide (2OH-FTA)	Anticancer drug	[35]
	Bicalutamide (BIC)	Anticancer drug	[36]
Agrochemical	Lindane (βHCH)	Pesticide	[37,40]
	Permethrin (PERM)	Insecticide	[44,45]
	Mancozeb (MNZ)	Fungicide	[38,39,41]
	Tributyltin oxide (TBTO)	Biocide/antifouling agent	[46]

**Table 2:** RCR values measured in human spermatozoa following exposure to agrochemical EDCs. The table reports RCR values obtained after treatment with βHCH, PERM, MNZ, and TBTO, together with those measured for the reference herbicides GLY and GA, across the different concentrations tested.

Compound	0.1 nM	1 nM	10 nM	100 nM	1000 nM
GLY	2.24 ± 0.34	2.08 ± 0.27	2.08 ± 0.09	2.00 ± 0.07	1.77 ± 0.20
GA	1.72 ± 0.36	1.69 ± 0.12	1.77 ± 0.38	1.61 ± 0.05	1.46 ± 0.02
βHCH	1.84 ± 0.09	1.71 ± 0.07	1.87 ± 0.45	1.56 ± 0.25	1.40 ± 0.05
PERM	1.08 ± 0.16	1.08 ± 0.03	1.02 ± 0.12	1.09 ± 0.05	1.01 ± 0.04
MNZ	1.07 ± 0.11	1.09 ± 0.07	1.01 ± 0.07	1.05 ± 0.09	1.07 ± 0.09
TBTO	1.09 ± 0.08	1.04 ± 0.04	1.04 ± 0.13	1.04 ± 0.04	1.02 ± 0.08

## Data Availability

The original contributions presented in this study are included in the article. Further inquiries can be directed to the corresponding author.

## Abbreviations

The following abbreviations are used in this manuscript:

2OH-FTA	dihydroxyflutamide
ADP	adenosine diphosphate
ANOVA	analysis of variance
ATP	adenosine triphosphate
BIC	bicalutamide
BSA	bovine serum albumin
CASA	computer-assisted sperm analysis
CCCP	carbonyl cyanide 4-chlorophenyl hydrazone
DDT	dichlorodiphenyltrichloroethane
DEHP	di-2-ethylhexyl phthalate
DMSO	dimethyl sulfoxide
EDCs	endocrine-disrupting chemicals
EDTA	ethylenediaminetetraacetic acid
GA	glufosinate ammonium
GLY	glyphosate
MNZ	mancozeb
NADH	nicotinamide adenine dinucleotide reduced
OXPHOS	oxidative phosphorylation
PERM	permethrin
RCR	respiratory control ratio
ROS	reactive oxygen species
SCA^®^	sperm class analyzer
SD	standard deviation
TBTO	tributyltin oxide
V_3_	mitochondrial oxygen consumption in the active respiratory state
V_4_	mitochondrial oxygen consumption in the resting respiratory state
WHO	World Health Organization
βHCH	lindane
